# Health and Economic Impact of Surgical Site Infections Diagnosed after Hospital Discharge

**DOI:** 10.3201/eid0902.020232

**Published:** 2003-02

**Authors:** Eli N. Perencevich, Kenneth E. Sands, Sara E. Cosgrove, Edward Guadagnoli, Ellen Meara, Richard Platt

**Affiliations:** * Beth Israel Deaconess Medical Center, Boston, Massachusetts, USA; † Centers for Disease Control and Prevention Eastern Massachusetts Prevention Epicenter, Boston, Massachusetts, USA; ‡ Harvard Medical School, Boston, Massachusetts, USA; § Brigham and Women’s Hospital, Boston, Massachusetts, USA

**Keywords:** surgical wound infection, infection control, medical economics, utilization, research

## Abstract

Although surgical site infections (SSIs) are known to cause substantial illness and costs during the index hospitalization, little information exists about the impact of infections diagnosed after discharge, which constitute the majority of SSIs. In this study, using patient questionnaire and administrative databases, we assessed the clinical outcomes and resource utilization in the 8-week postoperative period associated with SSIs recognized after discharge. SSI recognized after discharge was confirmed in 89 (1.9%) of 4,571 procedures from May 1997 to October 1998. Patients with SSI, but not controls, had a significant decline in SF-12 (Medical Outcomes Study 12-Item Short-Form Health Survey) mental health component scores after surgery (p=0.004). Patients required significantly more outpatient visits, emergency room visits, radiology services, readmissions, and home health aide services than did controls. Average total costs during the 8 weeks after discharge were US$5,155 for patients with SSI and $1,773 for controls (p<0.001).

Surgical site infections (SSIs), the second most common cause of nosocomial infection after urinary tract infections, cause approximately 17% of all hospital-acquired infections (*1*) and lead to increased costs and worse patient outcomes in hospital inpatients (*2*). The Centers for Disease Control and Prevention estimates that approximately 500,000 SSIs occur annually in the United States (*3*). Costs and outcomes secondary to SSIs can vary by location and surgery type. Infections in cardiac surgery have been estimated to add from US$8,200 (1982 dollars) to $42,000 (1985 dollars) to the cost of care after adjustments are made for preexisting illnesses and conditions, and these increased costs are likely attributable to excess hospital and intensive care unit stays (*4*–*6*). Overall, SSIs may result in $1–$10 billion in direct and indirect medical costs each year (*3*,*7*).

With the current trends favoring a shortened postoperative hospital stay, outpatient surgery, and same-day surgery, more SSIs are occurring after discharge from the hospital and, therefore, beyond the reach of most hospital infection control surveillance programs (*8*). Of all surgical procedures, 75% are now estimated to occur in the outpatient or ambulatory setting, and for those that do occur in the inpatient setting, postoperative length of stay is decreasing (*9*). An estimated 47% to 84% of SSIs occur after discharge; most of these are managed entirely in the outpatient setting (*8*,*10*).

Given the high costs and adverse patient outcomes associated with SSIs, quantifying the clinical and economic impact of SSIs recognized after discharge from the hospital is important. Several studies have focused on the direct medical costs borne by the hospital or insurer, but to our knowledge, no study has assessed the full societal impact of SSIs, which includes indirect costs, such as lost patient productivity and diminished functional status (*11*,*12*). Additionally, no study has addressed the costs of SSIs that arise from most of these infections which now occur in the postdischarge setting and for which patients are not readmitted to the index hospital. The magnitude of these costs might not be known if ascertainment were left solely to the index hospital’s information systems.

## Methods

This study used a matched cohort design to compare the costs and illness of patients with an SSI to matched patients who had surgery during the same period but in whom an SSI did not develop. The study population was drawn from adult members of Harvard Vanguard Medical Associates, a 250,000-member multispecialty group practice, which at the time of the study was a staff model component of Harvard Pilgrim Health Care, a health maintenance organization. Study participants were those who had undergone a nonobstetric inpatient or outpatient operating room procedure at Brigham and Women’s Hospital from May 18, 1997, through October 31, 1998. Cases of SSI were identified prospectively by using an established method of automated medical record screening for 102 diagnostic, testing, or treatment codes that may have indicated the occurrence of an SSI in the outpatient setting (*13*). In addition, pharmacy records were screened for antibiotic dispensing, and claims were screened for hospital readmissions or emergency room visits pertaining to an SSI. Surgeries were identified in 2-week cycles, and a total of 38 cycles were completed. An investigator reviewed those records judged to indicate a postdischarge SSI by initial screening, using the National Nosocomial Infections Surveillance criteria during the 30-day postoperative period to confirm infection (*14*). Patients who had an SSI that occurred during the index hospitalization were excluded. Case-patients were individually matched on surgery type, age and duration of surgical procedure in a ratio of one case-patient to two other members of the cohort.

### Questionnaire

Participants were enrolled 5–7 weeks after surgery. All case-patients and matched pairs were mailed a 49-item questionnaire, an explanatory letter, and a consent form. The questionnaire contained three sections. The first section had questions designed to assess illness, which were taken from the National Health Interview Survey, and additional questions designed to quantify care and resource use during the 8-week postoperative period, including home visits, phone calls to practitioners, missed days from work, and family members’ missed days from work (*15*). The second and third sections were each designed to assess health-related quality of life by using the Medical Outcomes Study 12-Item Short-Form Health Survey (SF-12) during the 8 weeks after surgery and the 4 weeks before surgery, respectively (*16*). Patients were instructed to recall their overall health since surgery and their health before surgery. Patients who did not return questionnaires were followed up with phone calls and re-mailing of the survey. If they did not return the questionnaire within 90 days, they were considered nonresponders. If questionnaires were incomplete, the answers that were provided were included in the analyses. SF-12 mental and physical scores (MCS-12 and PCS-12, respectively) were normalized by using standard methods to obtain mean scores (*16*).

### Administrative Databases

Four administrative databases were used to determine provider-level resource use associated with the 8 weeks after discharge from the operation that led to entry into the cohort. The Harvard Pilgrim Health Care demographic database was used to capture patient date of birth, gender, and zip code. This health maintenance organization maintains an automated administrative claims system that houses all charges from vendors, including hospitals, and outside the ambulatory-care centers. This database included the associated discharge date for index surgery, from which we calculated the 8 weeks’ postoperative time window for our analysis and from which we counted the resource utilization across all databases. This database provided all charges between the vendor or facility and the health maintenance organization, length of stay, procedure codes, diagnosis codes, and pharmacy codes for all encounters that occurred outside of the health plan. Thus, any readmission, emergency room visit, skilled nursing facility stay, or home health aide charge appeared in this database.

In addition, Harvard Pilgrim Health Care maintained an automated ambulatory medical record system that captured all ambulatory encounters and orders at its health centers. This database allowed determination of the number of outpatient visits, telephone calls, and most laboratory tests. This database also captures the number of inpatient physician encounters made by the health maintenance organization’s patients. Costs associated with outpatient visits at the health centers were imputed by using the costs for CPT Codes 99213–99215 from the 1998 National Physician Fee Schedule Relative Value File (available from: URL: http://www.hcfa.gov/stats/pufiles.htm). The first visit for each case-patient with an SSI was assumed to be an established-patient visit lasting 40 minutes (CPT 99215), and the first visit for those without an SSI was assumed to last 25 minutes (CPT 99214). All subsequent visits for all patients were assumed to last 15 minutes (CPT 99213). Costs in 1998 for CPT codes 99213, 99214, and 99215 were $41.46, $62.74, and $99.06, respectively.

Harvard Pilgrim Health Care also maintains a database that captures all pharmacy prescriptions dispensed in the outpatient setting (*17*). This database provided the standard wholesale costs for all antibiotic prescriptions for the 8-week postoperative period.

Chronic disease scores, as a marker for patient preexisting conditions and illnesses, have been shown to be predictors of SSI and also of death, hospitalization, and resource utilization (*18*–*21*). The chronic disease score, as used here, is a method for controlling for preexisting conditions on the basis of patient age, gender, and recent history of drug dispensing. This score predicts for hospitalization (*22*) and SSI (*19*) and thus would appear to be a useful adjuster for preexisting conditions in our cost analysis. For each patient, a chronic disease score was created by using patient age, sex, and presence or absence of 29 chronic diseases, calculated from the 6-month preoperative ambulatory pharmacy dispensing record (*18*,*19*).

Attributable charges of SSI recognized after discharge were calculated by taking the mean charges of case-patients and subtracting the mean charges of control patients. Mean charges were chosen for this comparison since the use of medians would negate the effect that even a moderately rare event (those that occur in <50% of the study population) would have on health-care costs. For those areas of resource utilization in which only charges were available, charges were converted to costs by using a cost-to-charges ratio. Since this study involved readmission and resource utilization at several different hospitals, conversion to costs would have required institution-specific ratios of costs to charge, to which we did not have access. We have, therefore, chosen to use a published ratio of costs to charges from a cohort of 4,108 patients admitted in the same city to two hospitals, one of which was the index hospital in this study, and during a similar period to this study (*23*).

### Statistics

Student t test, Wilcoxon rank-sum test, or Fisher exact test were used, where appropriate, for univariate comparisons. Outcomes are presented as medians with interquartile range, means with standard deviations, or proportions. Cases and matched controls were compared by using the Wilcoxon signed-ranks test for continuous outcomes with non-normal distributions, continuous linear regression by forcing the matching variable into the model for normally distributed variables, or the Cochran-Mantel-Haenszel for matched binary variables. Almost all assessed utilization outcomes, including all charges, were non-normally distributed so both medians with interquartile range and means with standard deviation are reported. Multivariable unconditional logistic regression was used to control for confounding variables in the analysis of the questionnaire data, and all matched variables were forced into the model to account for the matching process.

Since combined total costs and charges (ambulatory, pharmacy, and nonambulatory) of the entire cohort of 267 patients were log-normally distributed, the total cost variable was analyzed by using a log-transformation of total costs in a matched linear regression model. To estimate the effect that preexisting conditions or index surgery duration might have on the attributable effect of SSI on total costs, a matched linear regression with log-transformed total costs as the outcome was created with the predictors SSI/no SSI, chronic disease score (CDS), and index surgery duration entered as variables into the model. Results are given as β-estimates of effect, R-square statistic, and p value for five models (only SSI versus no SSI; only CDS; both SSI versus no SSI and CDS; both SSI versus no SSI and index surgery duration; and all three variables: SSI, CDS, and duration of index surgery). All statistical tests were two-tailed; p <0.05 was considered statistically significant. Statistical analyses were performed with SAS v 8.01 for Windows (SAS Institute, Inc., Cary, NC).

During the anticipated study period, 3,000 surgeries would be estimated to be performed and, given a 2.8% risk for infection beginning after discharge from the hospital (based on our prior observations), 84 SSIs would be recognized after discharge. This gave a power of 0.89 to detect >5 days lost from usual activities. Our actual sample of SSIs recognized after discharge was 89 (1.9%) from a sample of 4,571 procedures.

All data collected were combined into one dataset for final analysis, after which all unique identifiers were removed. In addition, each patient provided a signed consent form before completing the questionnaire and being enrolled in the study. The Harvard Pilgrim Health Care institutional review board approved this study.

## Results

SSI recognized after discharge was confirmed in 89 (1.9%) of 4,571 procedures. One hundred seventy-eight patients with similar age, procedure types, and surgical duration were matched to the SSI patients in a ratio of one case-patient to two controls (Table 1). No significant differences in age, gender, or surgery type between case-patients and matched controls were noted. Surgery duration was significantly longer for SSI patients, despite having been matched for procedure duration. This was expected because procedure duration is an important risk factor for infection.

**Table 1:** Descriptive characteristics of cohort in study of surgical site infections (SSI), Harvard Pilgrim Health Care, 1997–1998^a^

Characteristic	Case-patients N (% or SD^a^)	Controls N (% or SD^a^)	p value
Study cohort N=267	89	178	
**Demographics of complete cohort**			
Age (yr)	55.8 (+/-14.6)	57.5 (+/-13.3)	0.33^b^
Male gender	43 (48.3)	94 (52.8)	0.52^c^
Surgery duration (min)	177 (+/-112)	137 (+/-74)	0.037^d^
Chronic disease score	3,058 (+/-2636)	2,148 (+/-2285)	0.005^d^
Surgery location (inpatient)	73 (82)	149 (83.7)	1.0^c^
Surgery type			
Cardiac	26 (29.2)	53 (29.8)	1.0^c^
General	25 (28.1)	53 (29.8)	0.89^c^
Gynecology	2 (2.3)	4 (2.3)	1.0^c^
Neurology	4 (4.5)	8 (4.5)	1.0^c^
Orthopedic	15 (16.9)	32 (18)	0.87^c^
Other	2 (2.3)	3 (1.7)	1.0^c^
Plastic	5 (5.6)	6 (3.4)	0.51^c^
Urology	3 (3.4)	6 (3.4)	1.0^c^
Vascular	7 (7.9)	13 (7.3)	1.0^c^
**Description of questionnaire responders**			
Responder N=173 (65%)	50 (56.2)	123 (69.1)	0.042^c^
Age (yr)	57.3 (+/-13.7)	58.6 (+/-12.4)	0.54^b^
Male gender	25 (50)	69 (56.1)	0.50^c^
Surgery duration (min)	185 (+/-142)	144 (+/-81)	0.19^d^
**Surgery type**			
Cardiac	16 (32)	39 (31.7)	1.0^c^
General	18 (36.0)	35 (28.5)	0.37^c^
Gynecology	1 (2.0)	3 (2.4)	1.0^c^
Neurology	1 (2.0)	6 (4.9)	0.67^c^
Orthopedic	7 (14.0)	23 (18.7)	0.51^c^
Other	1 (2.0)	0 (0.0)	0.29^c^
Plastic	2 (4.0)	3 (2.4)	0.63^c^
Urology	1 (2.0)	4 (3.3)	1.0^c^
Vascular	3 (6.0)	10 (8.1)	0.76^c^
**Occupation (could check >1)**			
Employed	26.6%	30.8%	0.61^c^
Homemaker	29.8%	28.2%	0.85^c^
Retired	42.9%	61.5%	0.07^c^
Student	2.1%	2.5%	1.0^c^
**Preexisting medical conditions^e^**			
Congestive heart failure	12.2%	2.5%	0.018^c^
Diabetes	24.5%	11.5%	0.057^c^
Arthritis	38.8%	21.5%	0.034^c^

^a^Results are shown as no. (%) or mean +/- standard deviation, along with p value for comparison of cases with SSIs to controls without SSIs.

^b^Student t test.

^c^Fisher exact test.

^d^Wilcoxon rank-sum test.

^e^Thirteen additional preexisting conditions were assessed, including chronic lung disease, vision or hearing impairment, asthma, peptic ulcer disease, chronic back pain, hypertension, angina, myocardial infarction, stroke, kidney disease, and cancer; all were not significantly different between cases and controls with p>0.05.

### Impact on Health, Activities, and Perceived Care Needs

One hundred seventy-three (65%) of 267 questionnaires were returned. Those who completed the questionnaire (responders) were slightly older than those that did not respond (58.2 years vs. 54.6 years, p=0.05). No other differences between questionnaire responders and nonresponders were significant (Tables 2 and 3). Among patients who completed the questionnaire, no differences between case-patients and controls were significant for age, sex, and procedure types (Table 1), or in the baseline SF-12 assessment of mental and physical health (Table 3). Reported occupations of patients and controls did not differ, and few differences between case-patients and controls existed with respect to self-declared differences in pre-existing medical conditions (Table 1). Case-patients did experience longer duration of surgery than did controls. Case-patients were also more likely than controls to report a history of congestive heart failure (12% vs. 2.5%, p=0.02) and arthritis (39% vs. 22%, p=0.03). There was a trend towards more case-patients having diabetes than controls (24% vs. 12% p=0.06).

**Table 2:** Comparison of questionnaire responders to nonresponders, surgical site infection (SSI) study^a^

Characteristic	Responder N (% or SD^a^)	Nonresponder N (% or SD^a^)	p value
Study cohort N=267	173	94	
Demographics			
Age (yr)	58.2 (+/- 12.7)	54.6 (+/-15.2)	0.05^b^
Male gender	94 (54.3)	43 (45.7)	0.20^c^
Surgery duration (min)	152 (+/-91)	139 (+/- 98)	0.14^d^
Surgery type			
Cardiac	55 (31.8)	24 (25.5)	0.33^c^
General	53 (30.6)	25 (26.6)	0.57^c^
Gynecology	4 (2.3)	2 (2.1)	1.0^c^
Neurology	7 (4.1)	5 (5.3)	0.76^c^
Orthopedic	30 (17.3)	17 (18.1)	0.89^c^
Other	1 (0.6)	4 (4.3)	0.054^c^
Plastic	5 (2.9)	6 (6.4)	0.20^c^
Urology	5 (2.9)	4 (4.3)	0.72^c^
Vascular	13 (7.5)	7 (7.5)	1.0^c^

^a^Results are shown as no. (%) or mean +/- SD, along with p value for comparison of cases with SSI to controls without SSI.

^b^Student t test.

^c^Fisher exact test.

^d^Wilcoxon rank-sum test.

**Table 3:** Univariate analysis of questionnaire respondents, surgical site infections (SSIs) study^a^

	Case-patient N (% or SD^a^) (N=50)	Control N (% or SD^a^) (N=123)	p value
**HRQOL with SF-12**			
Preoperative MCS-12	51.7 (+/-9.6)	51.5 (+/-9.9)	0.96^b^
Postoperative MCS-12	47.6 (11.6)	52.4 (+/-9.2)	0.025^b^
Preoperative PCS-12	41.1 (+/-12.7)	45.0 (+/-10.9)	0.058^b^
Postoperative PCS-12	33.9 (+/-10.0)	38.7 (+/-9.8)	0.003^b^
Change MCS-12 with surgery	–4.1 (+/-11.0)	0.9 (+/-9.6)	0.004^b^
Change PCS-12 with surgery	–7.2 (+/-10.6)	–6.3 (+/-13.3)	0.67^b^
**Additional questions**			
Time and productivity costs			
If employed, missed work	66.7%	62.3%	0.81^c^
Average no. missed days at work	61.2 (+/-38.6)	57.5 (+/-40.6)	0.95^c^
Unable to do regular activities	60.6%	69.5%	0.39^c^
Missed activities, in bed >1/2 day	63.6%	41.8%	0.043^c^
Average no. days missed activities	49.6 (+/-41.3)	50.1 (+/-42.0)	0.90^d^
**Additional costs**			
Provider made home visits	69.4%	47.5%	0.011^c^
Could have used home visits	30.8%	12.8%	0.068^c^
Used paid housekeeper	6.3%	5.8%	1.0^c^
Used 24-hr hotline	12.2%	5.7%	0.20^c^
Could have used 24-hr hotline	21.4%	8.9%	0.052^c^

^a^Results are shown as mean (+/- SD) or % of total responders , along with p value for comparison of cases with SSIs to controls without SSIs. Abbreviations used: HRQOL, Health Related Quality of Life; SF-12, Medical Outcomes Study 12-Item Short-Form Health Survey; MCS, Mental Health Component Score of SF-12; PCS, Physical Health Component Score of SF-12.

^b^Student t test.

^c^Fisher exact test.

^d^Wilcoxon rank-sum test.

In assessing time and productivity costs, we found that case-patients (64%) were more likely than controls (42%) to have spent at least 1/2 day in bed, thus missing planned regular activities (p=0.04). However, differences between case-patients and controls in other areas of lost productivity, such as missed days of work and inability to complete regular activities, were not significant.

Case-patients with an SSI (69%) were more likely than controls (48%) to require home health provider visits (p=0.01). Similar results were found after controlling for age, procedure duration, and baseline SF-12 physical function. There were trends for patients with SSI wanting more home health visits than were provided and wanting a 24-hour hotline to contact a health-care practitioner. Patients, but not controls, reported significantly lower physical health and mental health component scores on the SF-12 after surgery, compared to their own baselines (p=0.003 and p=0.02, respectively).

### Health Resource Use in 8 Weeks after Surgery

Patients with SSI recognized after discharge required significantly more resources within the outpatient setting than those without SSI (Table 4). Significantly more patients with SSI had at least one ambulatory-care visit, and their average number of visits (7.5) was more than twice the average of those without SSI (3.4). Additionally, case-patients were significantly more likely to call their provider and to make more phone calls to their provider than controls. The number of laboratory tests ordered did not differ between cases and controls. Estimated ambulatory outpatient visits costs generated were on average $365 per case with an SSI and $160 per control during the 8-week postoperative period (p<0.001).

**Table 4:** Univariate analysis of 8-week postoperative resource utilization, surgical site infections (SSIs) study^a^

	Cases N=89		Controls N=178		
	Medians or proportions	Means	Medians or proportions	Means	p value
**Outpatient visit use**					
Required outpatient visit	85 (96)		153 (86)		<0.001^b^
Outpatient visits per patient	5 [4, 9]	7.5 (+/-6.3)	3 [1, 5]	3.4 (+/-3.0)	<0.02^c^
Estimated outpatient visit costs	$265 [$223, $430]	$365 (+/-264)	$146 [$63, $229]	$160(+/-128)	<0.001^c^
Lab test ordered by provider	69 (78)		143 (80)		0.66^b^
No. of lab tests ordered	1 [1, 3]	2.1 (+/-2.5)	1 [1, 2]	2.0 (+/-2.3)	0.58^c^
Patient phoned provider	77 (87)		125 (70)		0.002^b^
No. of phone calls made	3 [2, 6]	4.7 (+/-4.8)	1 [0, 4]	3.0(+/-3.8)	0.00c
**Pharmacy use**					
Standard wholesale costs for antibiotics per patient	$34.2 [$78.6, 10.6]	$60 (+/-71.6)	$0 [$0, $0]	$13.6(44.2)	<0.001^c^
**Emergency room use**					
Patient visits to emergency room	28 (31)		16 (9)		<0.001^b^
Emergency room charges per patient	$0 [$0, $370]	$333 (+/-729)	$0 [$0, $0]	$114 (+/-470)	<0.001^c^
**Radiology services use**					
Patients who had a radiologic test	36 (40)		49 (28)		0.023^b^
Radiology charges per patient	$0 [$0, $242]	$1,076 (+/-3,845)	$0 [$0, 124]	$587 (+/-2365)	0.022^c^
**Rehospitalization**					
Patients rehospitalized	30 (34)		21 (12)		<0.001^b^
Total rehospitalization charges	$0 [$0, $4,370]	$7,925 (+/-22,321)	$0[$0, $0]	$2,079 (+/-11,222)	<0.001^c^
Total rehospitalization costs	$0 [$0, $1,924]	$3,489 (+/-9,827)	$0[$0, $0]	$916 (+/-4,941)	<0.001^c^
Visited by provider in hospital	46(52)		61(34)		0.008^b^
Inpatient provider visits	1 [0, 6]	3.5 (+/-4.5)	0 [0, 3]	2.2 (+/-5.3)	<0.001^c^
**Skilled nursing facility use**					
Skilled nursing facility used	8 (9)		8 (4.5)		0.09^b^
Days in skilled nursing facility	0 [0, 0]	0.21 (+/-0.83)	0 [0, 0]	.21 (+/-1.8)	0.97^c^
Skilled nursing charges per patient	$0 [$0, $0]	$460 (+/-2,198)	$0 [$0, $0]	$204 (+/-1,651)	0.14^c^
**Home health aide use**					
Home health aide used	55 (62)		84 (47)		0.009^b^
Home health charges per patient	$110 [$0, $605]	$827 (+/-1,765)	$0 [$0, $275]	$579 (+/-2,812)	0.007^c^
**Durable equipment use**					
Durable medical equipment used	33 (37)		39 (22)		0.008^b^
Durable medical charges per patient	$0 [$0, $102]	$123 (+/-436)	$0 [$0, $0]	$69 (+/-223)	0.013^c^
Total costs**^d^**	$1,240 [$445, $4,594]	$5,155 (+/-10,8570	$300 [$146, $795]	$1,773 (+/-6,344	<0.001^c^

^a^Results are shown as no. (%), mean (+/- standard deviation) or median [interquartile range] along with p value for comparison of cases with SSI to controls without SSI.

^b^Cohran-Mantel-Haenszel.

^c^ Wilcoxon signed-ranks test.

^d^Total costs encompass all emergency, radiology, readmission, skilled nursing, home health, and durable medical charges that have been converted to costs with a cost-to-charge ratio and all estimated outpatient visit and antibiotic costs.

Patients with an SSI recognized after discharge also used significantly more resources outside of the ambulatory-care centers. More case-patients (31%) had at least one visit to an emergency room compared to controls (9%), p<0.001, and they generated significantly more emergency room charges ($333 vs. $114, p<0.001).

Those with SSI were more likely to require a radiology test (40% vs. 28%, p=0.02) and had higher radiology test charges ($1,076 vs. $587, p=0.02) than those without SSI. More patients with an SSI received durable medical equipment than did controls (37% vs. 22%, p=0.008) and generated higher average durable medical equipment–related charges ($123 vs. $69, p=0.01). A greater proportion of case-patients (62%) than controls (47%) required home health services (p=0.009). Charges related to home health services were higher for those with an SSI ($827) than for those without an SSI ($579), p=0.007. Twice as many case-patients required a stay in a skilled nursing facility (9% vs. 4.5%, p=0.09). There was a nonsignificant trend towards higher average skilled nursing charges for case-patients ($460 vs. $204 p=0.14); however, the average number of days in a skilled nursing facility was the same for case-patients and controls.

Patients with an SSI recognized after discharge generated higher standard wholesale costs for antibiotics than did controls without an SSI. Case-patients had an average cost of $60 for antibiotics, while controls had costs of $13.60 per person (p<0.001). Patients with an SSI were more likely to be readmitted to the hospital (34%) than those without an SSI (12%), p<0.001. These rehospitalizations led to $7,925 charges per person with an SSI compared with charges of $2,079 for those without an SSI (p<0.001). After the conversion of charges to costs, an SSI diagnosed after discharge was associated with excess costs of $2,573 ($3,489 minus $916) from rehospitalization across the entire population who developed an SSI, regardless of readmission status.

Total estimated costs per person incurred during the 8 weeks after discharge from the hospital associated with the index procedures were $5,155 for case-patients with SSI and $1,773 for controls without an SSI (p<0.001). Therefore, costs were $3,382 or 2.9 times greater in patients with SSI recognized after discharge. The subsets of these costs that occurred in those 216 patients never readmitted to any hospital (including the index hospital) were, on average, $928 in case-patients and $621 in controls (p<0.001). Therefore, patients with SSI had on average $307 additional costs that would not have been captured by an infection control surveillance system limited to the inpatient setting. Additionally, in this particular cohort of patients, 23% of all re-admissions and 18% of all emergency room visits occurred at institutions other than the index hospital; such visits and admissions would not have been captured by standard inpatient infection control surveillance.

The mean chronic disease score was significantly higher among case-patients (3,058) than controls (2,148) (p=0.005), as expected on the basis of the higher prevalence of selected chronic diseases in those at risk for an SSI. To determine if preexisting conditions could account for some of the costs associated with SSI recognized after discharge, we used a matched linear regression model; the calculated chronic disease score was the predictor for log-transformed total costs (Table 5). Although the chronic disease score was a strong independent predictor of postoperative resource use, even in this matched cohort, it was not a meaningful confounder of the impact of SSI on resource utilization. The parameter estimate for being a case was 1.30 for log-transformed costs in the unadjusted model and 1.20 for log-transformed costs in the adjusted model when chronic disease score was included. This finding suggests that, even after preexisting conditions are adjusted for, SSIs recognized after hospital discharge are significantly associated with higher total costs.

**Table 5:** Results of five separate matched linear regression models with log-transformed total costs as the outcome variable, surgical sites infection (SSI) study

Model no.	Predictor variable	β parameter estimate	Standard error	p value	R^2^
1	SSI (case)	1.30	0.21	<0.001	0.492
2	Chronic disease score	0.00018	0.00006	0.002	0.095
3	SSI (case)	1.20	0.21	<0.001	0.507
	Chronic disease score	0.00012	0.00005	0.03	
4	SSI (case)	1.27	0.22	<0.001	0.499
	Index surgery duration	0.0017	0.0017	0.3	
5	SSI (case)	1.17	0.22	<0.001	0.514
	Chronic disease score	0.0001	0.00005	0.02	
	Index surgery duration	0.0018	0.0017	0.3	

Even though we matched case-patients and controls on duration of index surgery, patients with SSI recognized after hospital discharge had significantly longer duration of surgery. To measure if duration of index surgery could confound the total attributable costs of SSI recognized after hospital discharge, we used a matched linear regression model with duration of index surgery and SSI as predictors for log-transformed total costs. The addition of duration of index surgery into the model did not significantly confound the attributable impact that SSI had on higher total costs (Table 5).

## Discussion

SSIs recognized after discharge from the hospital were associated with significantly higher direct medical costs and indirect costs. With respect to direct medical costs, SSIs diagnosed after hospital discharge incurred significantly more attributable use of resources than matched controls in each of the following categories: outpatient visits, inpatient care, pharmacy, radiology, home health aide care, and durable medical equipment. When all sources of direct medical costs were combined, SSIs recognized after discharge were associated with $3,382 in excess costs over those without SSI. This difference was significant after preexisting conditions and index surgery duration were controlled for. Importantly, in the linear regression models (Table 5), SSIs recognized after discharge explained one-half the variation in total costs (R2=0.49), and this finding was not altered by the addition of chronic disease score or index surgery duration.

Direct medical costs have been postulated to be low in patients who do not require readmission after a postdischarge SSI has developed (*10*). When readmission costs attributable to SSI ($2,573) were subtracted from total costs attributable to SSI ($3,382), we found that the mean charge manifest outside of the inpatient hospital setting attributable to SSI recognized after discharge was $809. Therefore, 24% of costs attributable to the SSI recognized after discharge would typically occur beyond the cost accounting systems of most index hospitals in which the initial surgical procedure was performed. This 24% would be the minimum fraction of the costs missed if all readmissions occurred at the index hospital. In our study, 23% of the readmissions occurred at settings other than the index hospital. Therefore, approximately $1,409 (42%) of all costs attributable to SSI were unknown to the index hospital. Kirkland et al. found that patients with an SSI had an increased risk of readmission and death associated with SSIs recognized during the initial hospitalization (*11*). No patients in our study died during the 8-week postdischarge follow-up period.

The matched cohort-design has been associated with selection bias when stringent matching criteria prevent some cases of SSI from being included in the study analysis (*24*,*25*). Selection bias was not a factor in this study because all cases of SSI were included.

We recognize that we were unable to assess all societal costs of SSI, such as individual patient transportation costs. However, in addition to the direct medical costs, we found that patients with SSI recognized after discharge had a significant decline in the mental health component of the SF-12. The magnitude of this drop, compared to results for controls, was similar to one reported for those who have experienced their first myocardial infarction (*26*). Case-patients were also more likely to spend more than one-half day in bed, missing their regular activities. The economic impact of spending this extra time in bed, however, appears to be minimal since we found no significant differences in other measures of productivity. The indirect costs of lost time at work could not be determined in this cohort since fewer than one-third of respondents were employed at the time of the study. A similar magnitude of use of home health aide providers was reported in the questionnaire and in the electronic claims database. This correspondence provides some evidence that respondents were representative of the entire cohort. Although patients were not asked about their use of resources in the 4 weeks before surgery until weeks after the surgery took place, we have found that for scaled scores, such as the SF-12 used in this study, patients consistently reported similar results during the hospital stay and 3 months later (*27*).

We conclude that SSIs diagnosed after hospital discharge were associated with significant impairment of physical and mental health. These SSIs also incurred substantial excess resource utilization across the spectrum of health care. These findings support the need to prevent SSIs that occur after discharge.
